# Development of a Rapid Serological Assay for the Diagnosis of Strongyloidiasis Using a Novel Diffraction-Based Biosensor Technology

**DOI:** 10.1371/journal.pntd.0003002

**Published:** 2014-08-07

**Authors:** Brian J. Pak, Fabio Vasquez-Camargo, Evgeniya Kalinichenko, Peter L. Chiodini, Thomas B. Nutman, Herbert B. Tanowitz, Isabel McAuliffe, Patricia Wilkins, Paul T. Smith, Brian J. Ward, Michael D. Libman, Momar Ndao

**Affiliations:** 1 Axela, Inc., Toronto, Ontario, Canada; 2 National Reference Centre for Parasitology, Research Institute of the McGill University Health Centre, Montreal, Quebec, Canada; 3 Department of Clinical Parasitology, Hospital for Tropical Diseases, University College London Hospitals, London, United Kingdom; 4 London School of Hygiene and Tropical Medicine, London, United Kingdom; 5 Laboratory of Parasitic Diseases, National Institute of Allergy and Infectious Diseases, National Institutes of Health, Bethesda, Maryland, United States of America; 6 Department of Pathology Medicine, Albert Einstein College of Medicine, Bronx, New York, United States of America; 7 US Centers for Disease Control and Prevention, Atlanta, Georgia, United States of America; 8 J.D. MacLean Centre for Tropical Diseases, Department of Medicine, McGill University, Montreal, Quebec, Canada; Yale Child Health Research Center, United States of America

## Abstract

**Background:**

Strongyloidiasis is a persistent human parasitic infection caused by the intestinal nematode, *Strongyloides stercoralis*. The parasite has a world-wide distribution, particularly in tropical and subtropical regions with poor sanitary conditions. Since individuals with strongyloidiasis are typically asymptomatic, the infection can persist for decades without detection. Problems arise when individuals with unrecognized *S. stercoralis* infection are immunosuppressed, which can lead to hyper-infection syndrome and disseminated disease with an associated high mortality if untreated. Therefore a rapid, sensitive and easy to use method of diagnosing *Strongyloides* infection may improve the clinical management of this disease.

**Methodology/Principal Findings:**

An immunological assay for diagnosing strongyloidiasis was developed on a novel diffraction-based optical bionsensor technology. The test employs a 31-kDa recombinant antigen called NIE derived from *Strongyloides stercoralis* L3-stage larvae. Assay performance was tested using retrospectively collected sera from patients with parasitologically confirmed strongyloidiasis and control sera from healthy individuals or those with other parasitoses including schistosomiasis, trichinosis, echinococcosis or amebiasis who were seronegative using the NIE ELISA assay. If we consider the control group as the true negative group, the assay readily differentiated *S. stercoralis*-infected patients from controls detecting 96.3% of the positive cases, and with no cross reactivity observed in the control group These results were in excellent agreement (κ = 0.98) with results obtained by an NIE-based enzyme-linked immunosorbent assay (ELISA). A further 44 sera from patients with suspected *S. stercoralis* infection were analyzed and showed 91% agreement with the NIE ELISA.

**Conclusions/Significance:**

In summary, this test provides high sensitivity detection of serum IgG against the NIE *Strongyloides* antigen. The assay is easy to perform and provides results in less than 30 minutes, making this platform amenable to rapid near-patient screening with minimal technical expertise.

## Author Summary

A rapid and sensitive serodiagnostic assay for strongyloidiasis based on a 31-kDa recombinant antigen from *Strongyloides stercoralis* (NIE) was developed using a novel diffraction-based optical biosensor technology. Assay performance was tested using retrospectively collected sera from patients with parasitologically confirmed strongyloidiasis (n = 54) and control sera from healthy individuals (n = 7) or those with other parasitoses including schistosomiasis, trichinosis, echinococcosis or amebiasis (n = 40). If we consider the control group as the true negative group, the assay readily differentiated *S. stercoralis*-infected patients from controls detecting 96.3% of the positive cases, and with no cross reactivity observed in the control group. These results were in excellent agreement (κ = 0.98) with results obtained by an NIE-based enzyme-linked immunosorbent assay (ELISA). A further 44 sera from patients with suspected *S. stercoralis* infection were analyzed and showed 91% agreement with the NIE ELISA. This test provides high sensitivity detection of serum IgG against the NIE *Strongyloides* antigen. The assay is easy to perform and provides results in less than 30 minutes, making this platform amenable to rapid near-patient screening with minimal technical expertise.

## Introduction

Strongyloidiasis is a persistent human parasitic disease caused by the intestinal nematode, *Strongyloides stercoralis*. It is endemic in the tropical and subtropical regions of the world where sanitary conditions are poor, and is increasing in prevalence even in resource-rich settings due to widespread travel and migration [1]–[3]. Worldwide, strongyloidiasis is estimated to affect at least 370 million people [4]. The exact prevalence of strongyloidiasis is not known because in many tropical and subtropical countries *S. stercoralis* can infect up to 60% of the population [5]. The majority of infected individuals are either asymptomatic or display intermittent, subtle, non-specific clinical symptoms that do not come to medical attention. Moreover, due to the unusual ability of *S. stercoralis* to auto-infect, infection can be life-long with most patients remaining unaware of their infection [5], [6]. Immunosuppression in infected patients, particularly with corticosteroids, can lead to a hyper-infection syndrome with uncontrolled dissemination of larvae and an associated mortality of up to 80% if untreated [7]–[12].

Currently, several imperfect methods exist for diagnosing strongyloidiasis. Stool examination with microscopic identification of larvae is considered the gold standard diagnostic procedure [13], [14]), showing good specificity with experienced staff. However, because of low numbers of adult parasites and irregular larval output during chronic, asymptomatic disease, this method lacks sensitivity, with false negative results in up to 70% of proven infections [13], [15]–[17]. Diagnostic sensitivity can be improved by analyzing serial stool samples [14], [17]–[19], larval enrichment from fecal samples by Baermann or concentration methods, or by agar plate coproculture [13], [14], [18], [20]. However, these approaches are time consuming, require a fresh stool sample and special technical training, and still lack sufficient sensitivity since they rely on the presence of intermittently shed larvae in the stool. Immunological approaches for detecting parasite-specific antibodies in serum by indirect enzyme-linked immuosorbent assays (ELISA) are well-described [6], [13], [21]–[28]. Most employ crude *S. stercoralis* filariform larvae extract and achieve reasonably high diagnostic sensitivity (∼85%) but lower specificity because of cross-reactivity with other tissue helminth infections [6], [13], [29]. While generally effective and suitable for batch testing, ELISA-based tests require moderately-sophisticated laboratory facilities to perform, limiting their use in many regions where *S. stercoralis* is endemic.

To circumvent the cross-reactivity associated with crude larval extracts, focus has recently turned to the use of recombinant antigens for *Strongyloides* serodiagnostics. In 2002, Ravi and colleagues [30] identified a 31 kDa recombinant antigen from an *S. stercoralis* L3 cDNA library which they named NIE. An NIE-based immunoassay had excellent sensitivity and did not cross-react with samples from individuals with other parasitic infections [13], [28], [31].

**Figure 1:**
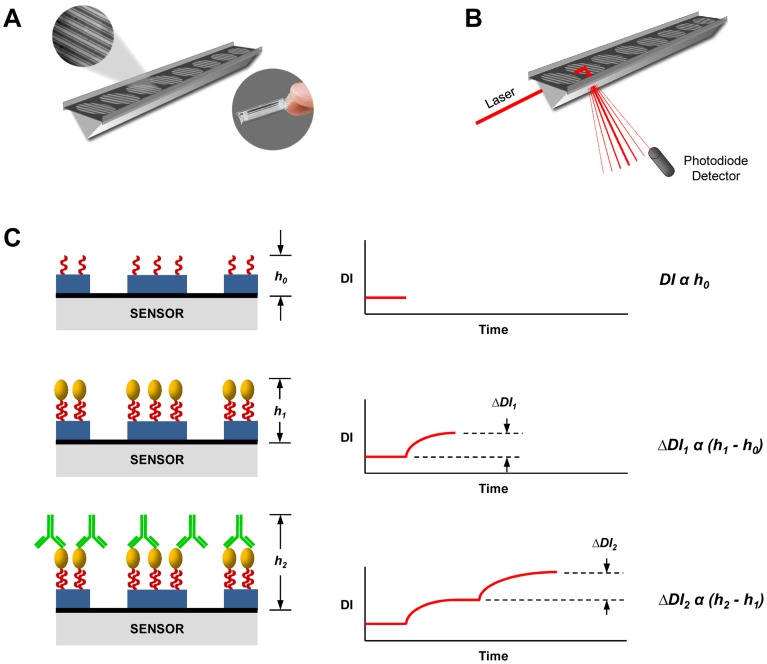
Schematic representation of a dotLab biosensor. (A) Each sensor consists of a contiguous array of 8 assay locations spotted on the bottom of a 10 µL flow channel where reagents and samples are introduced. Each assay location is comprised of a repeating pattern of capture molecules arranged in a defined series of parallel lines creating a diffraction grating. (B) Illumination of an assay spot with a laser generates a predictable diffraction image. The intensity of the diffraction image is monitored in real time by a photodiode detector. (C) Increases in the height (*h*) of the diffraction grating due to molecular binding events results in a proportionate increase in the diffraction image intensity (Δ*DI*).

In this report, we describe a rapid and high-sensitivity assay for *S. stercoralis* antibody using recombinant NIE antigen and a novel diffraction-based optical biosensor technology. The dotLab mX System (Axela, Inc., Toronto, ON) utilizes diffractive optics technology (dot) [32], [33] to provide label-free analysis of biomolecular interactions in real-time. Interactions occur in high precision, disposable, plastic biosensors which consist of a linear array of assay spots along the bottom of a 10 µL flow channel (Figure 1A). Each spot is comprised of capture molecules arranged in a defined pattern of parallel lines creating a diffraction grating. When illuminated with a laser, the grating generates a predictable diffraction image (Figure 1B) that increases in intensity as ligands bind. Changes in diffraction image intensity are monitored using a photodiode detector and yield real-time measurement of the binding events (Figure 1C). This approach combines the benefits of improved assay specificity using recombinant NIE with a robust platform that provides rapid, high-sensitivity results.

## Materials and Methods

### Ethics statement

Serum samples were obtained from multiple reference laboratories including the Canadian National Reference Centre for Parasitology (NRCP, Montreal, QC), University College London Hospitals (London, UK), the National Institutes of Allergy and Infectious Diseases (Bethesda, MD), the Centers for Disease Control and Prevention (Atlanta, GA) and the Albert Einstein College of Medicine (Bronx, NY) and were considered exempt. All samples used in this study were anonymized.

### Patient serum samples

Positive “gold standard” serum samples (confirmed stool positive for *S. stercoralis*; n = 54) were obtained from multiple reference laboratories. Negative control samples (n = 47) consisted of sera obtained from: 1) healthy individuals residing in Canada with no prior history of travel outside of Canada (n = 7); and 2) individuals with confirmed diagnosis of other parasitic infections including trichinosis (n = 8), filariasis (n = 9), schistosomiasis (n = 9), echinococcosis (n = 6) and amebiasis (n = 8), and were negative for *Strongyloides* by an ‘in-house’ NIE-based ELISA (NRCP). All of the selected control samples displayed very high ELISA optimal density (OD) in their assays for the respective antibody. A further 44 samples from patients with suspected *S. stercoralis* (i.e.: serology was requested by the treating physician) were obtained from the NRCP. All serum samples were stored at −80°C until use.

### Study design

Two sub-studies were performed: i) serum taken from patients with stool positive for *Strongyloides* vs negative controls who were healthy or had other parasites found. We used this retrospective study to set a threshold for the assay and derive the sensitivity and specificity estimates; ii) a second study was performed prospectively using serum from the same pool as well as 44 others with possible infection (i.e.: serology ordered by treating physician). This study evaluated the agreement between the NIE ELISA and the new test.

### Recombinant NIE antigen

NIE cDNA cloned into pET30b plasmid was generously provided by Dr. Franklin Neva (National Institutes of Health, Bethesda, MD). The plasmid was transformed into *E. coli* strain BL21 (DE3) and the NIE was isolated from insoluble inclusion bodies at the NRCP as previously described [30]. Briefly, the purified NIE protein contained a plasmid-encoded 52 amino acids including six His tags at its N-terminal. The NIE fusion protein was purified using the His Bind Kit (Novagen, Inc., Billerica, MA), concentrated by ultrafiltration and then run on a size exclusion column (Superdex 75 HiLoad 16/60; Amersham Pharmacia Biotech, Baie d'Urfe, QC) to remove high molecular weight contaminants. Following a series of dialysis reactions in decreasing concentrations of urea (5-0.25 M in 20 mM Tris-HCl, pH 7.5, 300 mM NaCl, 5 mM EDTA, 2 mM DTT) to allow slow renaturation, the eluates were dialysed against PBS for 4 hours.

### Oligonucleotide-based addressing system and NIE conjugation

The panelPlus oligonucleotide-based addressing system was used for NIE immobilization onto dotLab Sensors (Axela, Inc., Toronto, ON). This approach is based on oligonucleotide hybridization to target the immobilization of capture molecules to specific locations on dotLab Sensors. The system consists of complementary pairs of 30-bp oligonucleotides, one of which is used to tag capture molecules with the other pre-coated on the dotLab Sensors. Incubation of oligonucleotide-conjugated capture molecules in panelPlus Sensors (Axela, Inc., Toronto, ON) results in their immobilization onto the sensor surface. The panelPlus system allows for either replicate analysis of individual assays or multiplexing capabilities using sensors pre-coated with several different oligonucleotides. Recombinant NIE was conjugated to D oligonucleotides using the panelPlus Labeling Kit (Axela, Inc., Toronto, ON) following the manufacturer's recommended protocol. NIE conjugation was performed at roughly a three oligonucleotide to one NIE molar ratio.

### NIE dot assay

All assays were performed on the dotLab mX System using panelPlus D Sensors (Axela, Inc., Toronto, ON). These sensors are provided with a capture surface consisting of D anchor oligonucleotides complementary to the D oligonucleotides conjugated to recombinant NIE. Serological assays were performed using the panelPlus Serology Kit (Axela, Inc., Toronto, ON) with a running buffer of HEPES buffered saline containing 0.1% Tween-20 (HBST). Briefly, sensors were blocked with blocking buffer, followed by a two minute incubation with oligonucleotide D-conjugated recombinant NIE (NIE@D; 231 ng/assay) resulting in NIE immobilization on the sensor surface. The sensors were washed with running buffer, and then incubated for three minutes with a 1∶20 dilution of patient serum (3.5 µL of neat serum). Following a brief wash, antibody binding signal was amplified using a 1∶10 dilution of goat anti-human IgG antibody-coated 40 nm gold colloid (BioAssay Works, LLC., Ijamsville, MD). Assays were performed with three-spot monitoring yielding three separate serum antibody measurements per assay.

### NIE ELISA

All serum samples were also tested by an NIE ELISA that was developed and validated at the NRCP. In brief, 96-well microtiter plates (Immulon 2; Thermo Labsystems, Franklin, MA) were coated overnight at 4°C with recombinant NIE diluted in 18 mM Na_2_CO_3_, 45 mM NaHCO_3_, pH 9.6. Wells were washed four times with phosphate-buffered saline containing 0.05% Tween 20 (PBST) and then blocked using 100 µL of 2% BSA in PBST. One hundred microliters of diluted test sera (1∶200) was added to each of the wells and incubated for one hour at 37°C. Following four washes with PBST, 100 µL of horseradish peroxidase (HRP)-conjugated goat anti-human antibodies (1∶16,000 dilution; PerkinElmer, Waltham, MA) was added to the wells for 30 minutes. The wells were washed three times with PBST and then 100 µL of 3,3′,5,5′-tetramethyl-benzidine (TMB) substrate (Millipore Corp., Billerica, MA) was dispensed into each well. After 10 minutes, the reaction was stopped by the addition of H_2_SO_4_. The plates were read in a spectrophotometer at 450 nm. Samples with optical densities (OD)<0.2 were considered negative, OD≥0.2 but <0.3 were considered equivocal and OD≥0.3 were considered positive for *Strongyloides* infection.

### Data analysis

The amplitude of the 40 nm gold colloid binding signal (ΔGNP) was normalized to the amplitude of the NIE@D binding curve (ΔNIE) to account for slight variations in sensor surface binding capacity yielding a normalized diffractional intensity (nDI) value that is proportional to antibody titer. Binding curve amplitudes and nDI calculations were performed using the Quantitation Editor module in version 1.1.3.4 of the dotLab Software (rev 8170; Axela, Inc., Toronto, ON). Assays were performed with three-spot monitoring, generating three independent titer measurements. An exclusion criterion of ΔNIE<0.075 DI or ΔNIE>0.310 was used to omit spots within a sensor. Each serum sample was analyzed twice and the average of the duplicate assays was taken as the measurement of antibody titer. For sample classification, a cutoff of five standard deviations above the mean nDI of the control samples was taken as the diagnostic threshold.

### Statistical analysis

Assay variability was evaluated based on the replicate analysis (n = 5) of a *Strongyloides* positive serum sample performed with three spot monitoring. Intra-assay reproducibility was calculated as the average coefficient of variance (CV) of the three spots monitored per assay while inter-assay variability was as determined by the CV of the five assays. Comparisons between parasitologically proven *Strongyloides* and control groups were performed using the Mann-Whitney U test. A *p* value of ≤0.05 was considered statistically significant. Concordance between the NIE dot assay and NIE ELISA was determined using Cohen's kappa coefficient (κ).

## Results

### NIE dot-based serological assay

**Figure 2:**
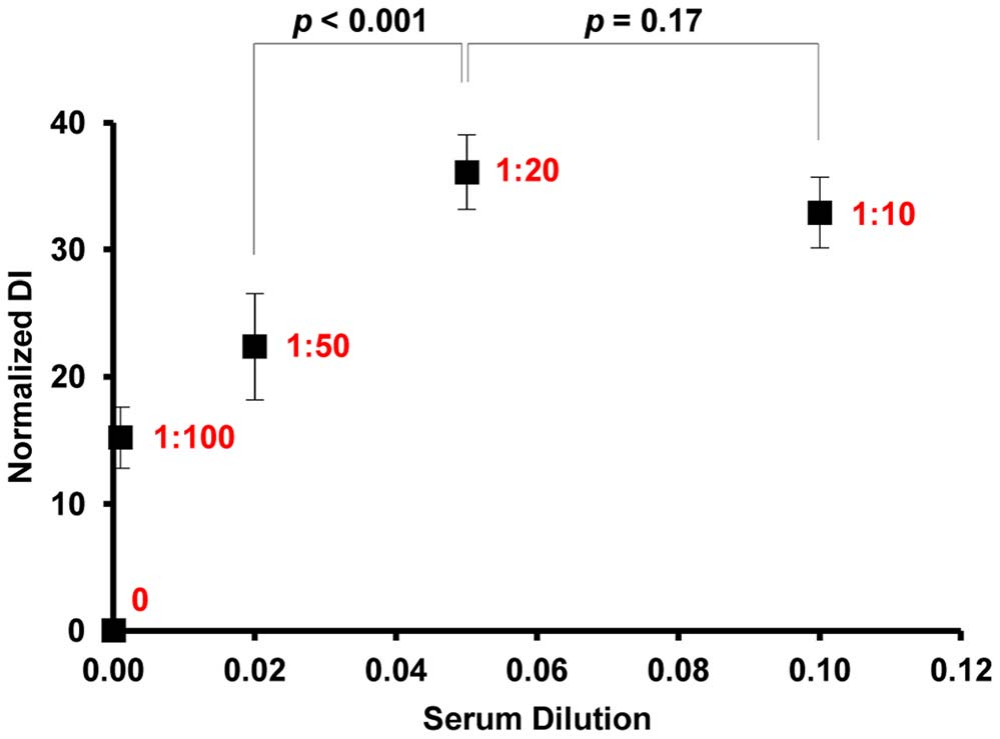
Serum dilution optimization. A series of different dilutions of *Strongyloides* positive serum were analyzed to determine the optimal serum concentration for use in dot-serology assays. Dilutions of 1∶10 and 1∶20 generated the highest antibody signal with no differences between the two dilutions (*p* = 0.17), while a significant decrease in signal intensity was observed between 1∶20 and 1∶50 dilutions (*p*<0.001). A serum dilution of 1∶20 was determined to be optimal for the dot-based *Strongyloides* assay. Data represent mean ± SD.

To determine the optimal serum concentration for antibody detection sensitivity, a series of serum dilutions were analyzed. A *Strongyloides* positive serum sample was tested at dilutions of 1∶10, 1∶20, 1∶50 and 1∶100. As shown in Figure 2, the highest antibody signal was obtained at serum dilutions of 1∶10 and 1∶20 with no difference in signal between the two dilutions (*p* = 0.17). However, a significant reduction in signal was observed between dilutions of 1∶20 and 1∶50 (*p*<0.001). Therefore, in order to obtain maximal signal at the highest possible dilution, a serum dilution of 1∶20 was used for all assays.

**Figure 3:**
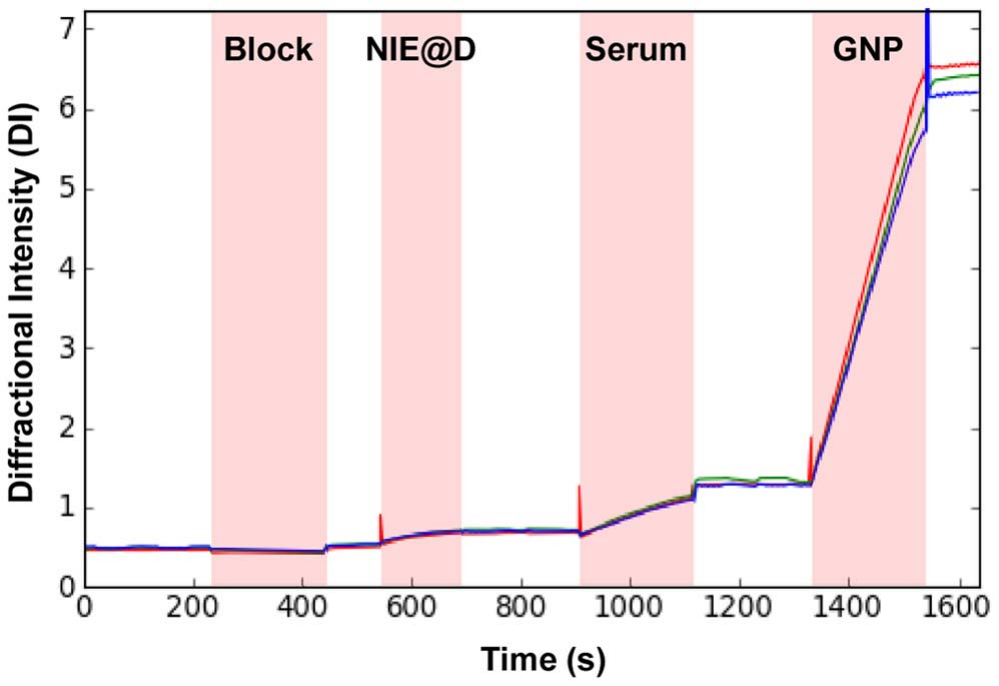
Representative trace of a dot-based serological analysis of a *Strongyloides* positive serum sample. The dotLab mX System outputs a real time trace displaying each reagent incubation and wash step in the assay. Note the binding curves representing the immobilization of NIE@D conjugate and serum anti-NIE antibodies. Significant signal amplification is achieved using anti-human IgG antibody conjugated gold nanoparticles (GNP). The three superimposed traces represent the results of a single assay performed with three spot monitoring.

Figure 3 represents a typical trace obtained from the analysis of a *Strongyloides* positive serum sample on the dotLab mX System. The regions of the trace highlighted in pink correspond to the incubation of each of the reagents used in the assay while the non-highlighted regions represent washes with running buffer. Sample delivery to the sensors, incubations and washes were fully automated on the dotLab mX System and total assay time was less than 30 minutes. Assay reproducibility was determined based on five replicate analysis of a pooled *Strongyloides* positive serum sample performed with three-spot monitoring. These results displayed good reproducibility with an intra-assay and inter-assay CV of 9.9% and 14.0% respectively.

### Diagnostic performance of NIE dot assay

**Figure 4:**
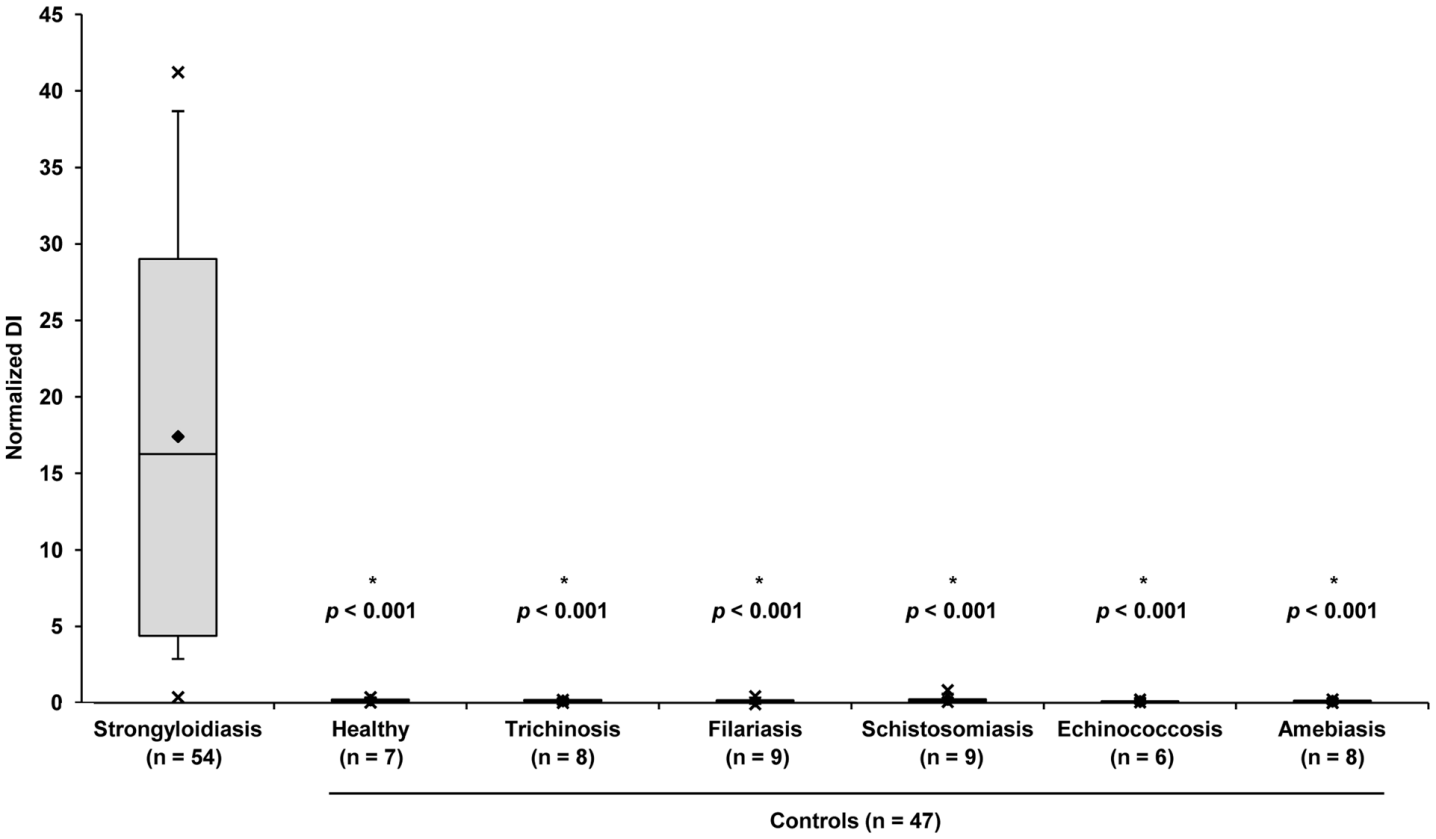
Box and whiskers plot of seven groups of sera tested for anti-NIE IgG antibodies. The plots summarize the results of gold standard *Strongyloides* samples (n = 54) and six control groups comprised of healthy individuals (n = 7) as well as those with trichinosis (n = 8), filariasis (n = 9), schistosomiasis (n = 9), echinococcosis (n = 6) and amebiasis (n = 8). The lower and upper boxes represent the samples in the second and third quartile respectively while the error bars above and below the box correspond to the 95^th^ and 5^th^ percentiles. The horizontal lines separating the boxes represent the median and the diamond denotes the mean. X represents the minimum and maximum values. A significant difference was observed between the gold standard *Strongyloides* and all control groups (*p*<0.001).

The dot-based *Strongyloides* serological assay using recombinant NIE was effective in distinguishing parasitologically proven *S. stercoralis* patients from controls. All 54 gold standard *Strongyloides* serum samples displayed a detectable antibody signal with an average normalized diffractional intensity (nDI) of 17.41 (range 0.36 to 41.22) compared with an average nDI of 0.14 (range −0.10 to 0.83) for the 47 control samples (*p*<0.001). As shown in Figure 4, a significant difference was found between the nDIs of the gold standard *Strongyloides* sera and each of the control groups: 1) healthy uninfected (n = 7; *p*<0.001); 2) trichinosis (n = 8; *p*<0.001); 3) filariasis (n = 9; *p*<0.001); 4) schistosomiasis (n = 9; *p*<0.0001); echinococcosis (n = 6; *p*<0.001); and amebiasis (n = 8; *p*<0.001).

To estimate assay sensitivity and specificity in this particular selection of specimens, a cutoff of 0.93 nDI representing the average nDI of the control samples plus five standard deviations, was used for sample classification. Based on this criterion, 52 of 54 gold standard sera were classified as positive, corresponding to a sensitivity of 96.3%. Assay specificity was 100% with all 47 control sera yielding signal below the cutoff.

### Comparison with NIE ELISA

The qualitative agreement between the NIE dot assay and NIE ELISA was determined by calculating the kappa coefficient (κ). Results for all gold standard and control samples showed almost perfect concordance between these two methods with κ = 0.98. The analysis of a further 44 samples from patients with suspected *S. stercoralis* infection also showed excellent agreement between NIE dot and ELISA with agreement in 40 of 44 (91%) samples. The four discordant samples were classified as *Strongyloides* positive by NIE ELISA but had OD values just above the defined equivocal threshold. There was no evidence of a pro-zone effect noted in samples with high antibody titers at the 1∶20 serum dilution used.

## Discussion

Due to the subclinical nature of most infections with *S. stercoralis* and its ability to auto-infect, strongyloidiasis is a persistent disease that can remain undetected for decades following initial exposure. With increasing use of corticosteroids and other immunosuppressive/immunomodulatory therapies for the treatment of a wide variety of disease states [12], [34]–[36], there is considerable cumulative life-time risk of release of *S. stercoralis* from immune control. Disseminated disease in these individuals can be associated with high mortality [12], [37]–[40]. Serological assays performed by enzyme-linked immunosorbent assay (ELISA) using crude extract of infective larvae have emerged as an alternative method of diagnosing *Strongyloides* infections [23], [25], [28], [29], [41]. Although relatively simple, these assays still have several limitations in regions where *Strongyloides* is endemic including availability, turn-around-time and the requirement for moderately-sophisticated laboratory infrastructure. For critically ill patients with hyper-infection syndrome and importantly, screening those at risk of harboring occult *Strongyloides* infection prior to their starting immunosuppression, a truly rapid test would have real advantages, though antibody may not be detectable in advanced immunosuppression [13], [42], [43].

In this study, we describe a rapid and simple serological assay based on real-time optical diffraction (NIE dot) that can accurately differentiate patients infected with *S. stercoralis* from both healthy individuals and those infected with other tissue parasitic infections. This assay clearly resolved infected individuals from controls with a 133-fold difference in average signal intensity. In this study, we defined the healthy subjects and other parasite controls group as the ‘true negative’ group and the subjects with stool positive *Strongyloides* group as a ‘true positive’ group. So, using a diagnostic cutoff of five standard deviations above the average signal obtained from all control samples, the NIE dot assay would have a sensitivity of 96.3% and specificity of 100% in this selected population. Although these results were based on a relatively small sample set, these findings represent a significant improvement over ELISAs based on crude *S. stercoralis* antigen which have reported sensitivity and specificity ranges of 83%–97% and 78%–98% respectively [6], [13], [23]–[26], [28], [41], [44]. Typically these studies showed a strong reciprocal relationship between better specificity at the expense of sensitivity or vice versa. The excellent performance of the NIE dot assay can be partially attributed to the use of recombinant NIE rather than crude antigen. Cross-reactivity of *Strongyloides* ELISAs based on crude larval antigens is common for subjects with other tissue helminth infections, particularly filariasis and schistosomiasis [23]–[26], [45], [46]. Consistent with our findings, other recent studies using immunoassays based on recombinant NIE had little cross-reactivity [28], [30], [31]. NIE-based assay formats such as ELISA, luciferase immunoprecipitation system (LIPS) and NIE dot have all reported >95% specificity while achieving >97% sensitivity, with the understanding that true negatives are difficult to define. In addition to reduced cross-reactivity, the use of recombinant proteins significantly simplifies the antigen preparation process. Crude *Strongyloides* antigen is produced from filariform larvae obtained from fecal cultures from heavily infected patients or experimental animals [47], [48]. This process is dangerous (L3 larvae are infective to humans), time-consuming and labor intensive as fecal samples need to be cultured for almost a week, then concentrated and purified to obtain suitable larvae for antigen preparation. Crude larval antigen is therefore difficult to produce reliably and in large quantities, leading to variation between antigen lots. The use of the recombinant NIE antigen therefore represents a significant advance in *Strongyloides* serodiagnosis.

This study is limited by the lack of control samples from patients infected with other intestinal parasites (e.g.: hookworms, *Ascaris lumbricoides*). This could theoretically lead to an overestimation of the specificity of dotLab NIE. However, in other hands, the NIE antigen has shown good specificity in this type of specimen [28]. As described by several authors and reviewed by Requena-Mendez [13], in immunosuppressed patients, the sensitivity of the ELISA might be lower. At the time this work was performed, we had access to only one sample from a patient with disseminated *Strongyloides* on which NIE ELISA was negative. Unfortunately the remaining sample was insufficient to be tested with the dotLab NIE. Further prospective study will be required to validate the performance of the NIE antigen in general and these immunocompromised subjects in particular.

The dotLab mX diffractive optics system used in this study offers a number of distinct advantages over conventional immunoassay platforms. The system is simple to operate and generates results in less than 30 minutes, making it amenable for more general distribution and near-patient settings. The panelPlus oligonucleotide-based addressing system used to immobilize recombinant NIE to the dotLab Sensors facilitates customization of multiplex assays. This system utilizes a library of unique 30-bp oligonucleotides, each of which can be conjugated to a different protein target. Using panelPlus sensors bearing a linear array of spots, each coated with different oligonucleotides complementary to those used for target conjugation, multiple antigens can be immobilized on a single sensor at user designated locations. The dotLab mX System interrogates each spot independently during an assay and yields multiple real-time traces representing molecular interactions that occur on each spot. Therefore, the dotLab mX System using panelPlus has the potential to perform multiplex assays in near-patient settings.

For *Strongyloides*, a number of different recombinant proteins in addition to NIE have previously been described as potential antigens for serodiagnostic use. These include 5a [49], 12A [49] and SsIR [31]. Multiplex serological assays using a combination or all of these antigens may provide improved assay performance over single antigen tests. Indeed, Ramanathan and colleagues recently showed that an LIPS assay using both NIE and SsIR improved overall assay performance compared to NIE alone [31]. Based on our work, multiplexing one or more *Strongyloides* antigens with antigens from other pathogens having similar clinical presentations on the dotLab mX System could serve as a rapid screening test in some settings. Lastly, *S. stercoralis* co-infection with human T cell lymphotropic virus type 1 (HTLV-1) is known to have important clinical implications. HTLV-1infection results in T cell proliferation leading to a shift from a Th2 to Th1 immune response and concomitant increase in interferon gamma (IFN-γ) and interleukin 10 (IL-10), and lower levels of interleukin 4 (IL-4), 5 (IL-5), 13 (IL-13) and parasite-specific IgE [50]–[52]. Co-infected patients are at much greater risk of developing disseminated strongyloidiasis [51], [52]. A multiplex *Strongyloides* assay that included HTLV-1 screening might improve the management of these patients.

In summary, we have developed a rapid, simple serodiagnostic assay for detecting *S. stercoralis* IgG using recombinant NIE antigen and a novel, high-sensitivity diffractive optics technology (dot). This assay performed as well as an NIE-based ELISA and LIPS. This platform generates results in less than 30 minutes and is fully automated requiring minimal user intervention, making it potentially attractive for near-patient testing and for use in regions where technical expertise or adequate laboratory facilities may not be available. With the ability to create custom multiplex assays using an oligonucleotide-based addressing system (panelPlus), the dotLab mX System could also be used further to improve *Strongyloides* serodiagnostics by incorporating multiple recombinant antigens in a multiplex format or by simultaneously screening for clinically relevant co-infections such as HTLV-1.

## Supporting Information

Checklist S1 STARD checklist.
(DOC)
